# Extent of Resection for Supratentorial Gliomas Using the B-mode Ultrasound as an Intraoperative Aid

**DOI:** 10.7759/cureus.72926

**Published:** 2024-11-03

**Authors:** Javier A Jacobo, Rafael Vazquez-Gregorio, Jorge Aristizabal, Diego Pineda, Andres F Cardona-Zorrilla

**Affiliations:** 1 Neuro-Oncology, Fundación Centro de Tratamiento e Investigación Sobre Cáncer Luis Carlos Sarmiento Angulo (CTIC), Bogotá, COL; 2 Neurosurgery, National Institute of Neurology and Neurosurgery, Mexico City, MEX; 3 Neurosurgery, Fundación Cardioinfantil, Bogotá, COL; 4 Neuroradiology, Fundación Centro de Tratamiento e Investigación Sobre Cáncer Luis Carlos Sarmiento Angulo (CTIC), Bogotá, COL

**Keywords:** extent of resection, glioma, intraoperative, surgery, ultrasound

## Abstract

Background: The cornerstone of glioma treatment is the surgical resection of the visible tumor, knowing fully that the disease extends beyond what magnetic resonance imaging (MRI) is able to show and the efficacy of the surgery is highly dependent on the surgeon's expertise. Different intraoperative technologies have emerged to aid in the goal of optimizing the extent of resection for glial tumors. Intraoperative ultrasound (iUS) is an attractive option due to its low cost and real-time imaging. This paper aims to illustrate the utility of this technology as an intraoperative aid for the resection of supratentorial gliomas.

Methods: This is an observational and retrospective study that included patients who received surgical resection for supratentorial gliomas using iUS as the only imaging intraoperative aid. Adult patients with supratentorial gliomas who were taken into surgery for tumor resection were included in the analysis. Demographic and clinical variables of the patients at the time of diagnosis were collected, including sex and age. Tumor morphological variables were collected, including the affected lobes, tumor volume, histological and molecular diagnosis, and extent of resection. The IBM SPSS Statistics for Windows, Version 27.0 (Released 2020; IBM Corp., Armonk, New York, United States) was used for the statistical analysis.

Results: A total of 44 patients were included in the analysis. Thirty-six patients (81.8%) had a high-grade glioma as the final diagnosis. The extent of resection achieved was over 90% in 70.5% of the cases.

Conclusion: iUS by itself has been proven to be a valuable tool to improve the localization and resection of supratentorial gliomas.

## Introduction

Glial tumors represent an important health issue worldwide with an estimated annual incidence of 6.6/100,000 individuals in the United States [1]. Despite aggressive multimodal treatment, the median overall survival (OS) for newly diagnosed high-grade glioma patients remains at about 18 months.

The cornerstone of glioma treatment and the first step is the surgical resection of the visible tumor, and its efficacy is highly dependent on the tumor grade and the surgeon's ability to adequately depict the tumor margins and excise the tumor completely [2]. It is important to note that the surgeon's knowledge of anatomy and their experience are among the most important factors. Moreover, additional intraoperative aids, including intraoperative ultrasound (iUS), serve as a tool to maximize these abilities. 

Different intraoperative technologies have emerged in recent years to aid in the goal of optimizing the extent of resection (EOR). These devices include different technologies such as neuronavigation systems, intraoperative computed tomography (iCT), intraoperative magnetic resonance imaging (iMRI), and iUS.

We aim to illustrate the utility of iUS as an intraoperative aid for the resection of supratentorial gliomas.

## Materials and methods

This is an observational and retrospective study that includes patients who were taken into surgery for the resection of supratentorial gliomas using iUS as the only imaging intraoperative aid. The institutional database was searched for adult patients with supratentorial gliomas who were taken into surgery for glioma resection from January 2021 to December 2023; these patients were included in the analysis.

Patients with tumors near eloquent areas of the brain received intraoperative cortical and subcortical mapping as an additional aid for the resection.

The ultrasonography system used during surgery for the localization and resection of the tumors was the Siemens ACUSON Redwood system (Erlangen, Germany), and a linear 10L4 transducer was used. All surgeries were performed by the senior author (JAJ) who has formal training in surgical neuro-oncology and has five years of experience using iUS as an intraoperative aid for the resection of glial tumors.

The technique for tumor resection begins with adjusting the default settings of the US device to obtain a better image during the procedure. The first step is to adjust the depth and image gain until the region of interest shows the best image quality possible. After choosing the adequate probe and the US setting is in place, the next step is to adequately place the transducer. The transducer should be held so that the external notch is facing the patient's anatomic right in a cross-sectional view or toward the head in a longitudinal view. By doing so, the surgeon can reliably assess where the lesion is displayed in the B-mode and take the necessary actions during the procedure. The scanning planes are similar to the familiar anatomical planes, and the position of the probe in relation to the surface of the brain will correspond to the plane in the preoperative images.

**Figure 1:**
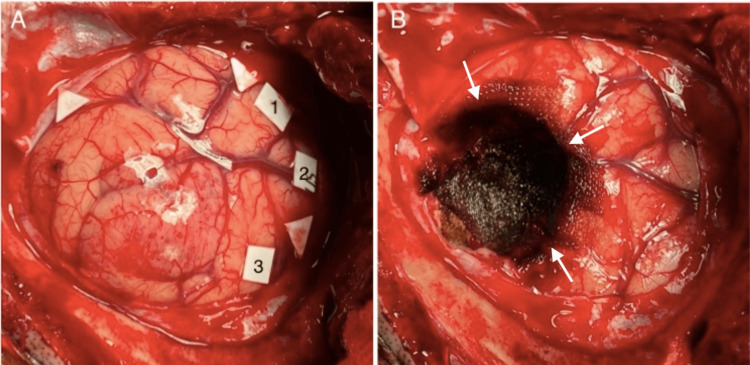
Intraoperative view of the cortical surface of the brain Arrowheads point to the margins of the tumor as viewed in the iUS. White squares indicate positive sites in the motor cortical mapping (A). The circular corticotomy (white arrows) was made along the lines of the tumor margins and a 1 cm margin for positive sites during the motor mapping (B). iUS: intraoperative ultrasound

The first step for tumor resection is to identify tumor boundaries in a circumference manner according to the obtained images during the iUS scanning. Together with cortical mapping, the tumor margins will guide the initial corticotomy to initiate tumor resection (Figure 1). The resection is carried out based on anatomical and functional margins until the deeper portion of the tumor is reached. After hemostasis, a final look at the resection cavity is performed with the US device. Care is taken to not misinterpret residual blood and hemostatic agents for a residual tumor. If a residual tumor is suspected at the margins of the resection cavity, further resection will take place. 

Demographic and clinical variables of the patients at the time of diagnosis were collected, including sex and age. Tumor morphological variables were collected, including the affected lobes, tumor volume, histological and molecular diagnosis, and EOR.

The pre- and postoperative tumor volumes were measured in cm^3^ on a presurgical and a 24-48h postoperative 3T MRI to establish the EOR. For high-grade gliomas, the volume measured was the T1 contrast enhancement region in the preoperative MRI, which was the objective of resection. For low-grade gliomas, the volume measured was the region of high signal intensity in the T2-weighted image, which was the objective of the resection for these tumors.

The percentage of tumor resection was determined by a neuroradiologist with over 20 years of experience using the 3D slicer software. 

We categorized the EOR into four categories: biopsy if the EOR was 60% or under, subtotal resection (STR) if the EOR was between 60% and 90%, near-total resection (NTR) if the EOR was between 90% and 99%, and finally gross total resection (GTR) if the EOR was 100% and over.

Tumor grade was determined using the neuropathology report of our institution based on the 2021 WHO classification. To determine tumor classification, different variables were taken into account which included IDH1 mutation status, p53 expression, and Ki-67 proliferation index via immunohistochemistry. The presence of IDH1 and IDH1/2 mutational testing was confirmed using immunohistochemistry for the IDH1 R132H variant. ATRX mutation status via immunohistochemistry was utilized to determine astrocytic lineage. 

The IBM SPSS Statistics for Windows, Version 27.0 (Released 2020; IBM Corp., Armonk, New York, United States) was used to determine measurements of central tendency in the sociodemographic and clinical variables (age, sex, diagnosis, tumor location, imaging characteristic, degree of resection, IDH and p53 mutation) using quantitative analysis measures for the said variable, mean, median, range, and standard deviation.

A categorization was carried out using distribution graphs for quantitative and qualitative variables, using the determination of the normality distribution with the Shapiro-Wilk test.

Inferential statistics were performed using the chi-squared and Spearman correlation tests for qualitative variables and parametric or non-parametric tests were used for quantitative variables depending on their distribution. A p-value less than 0.05 was considered to be statistically significant. 

## Results

A total of 44 patients were included in the analysis, with an average age of 45.48±15.95 years (19-81). The surgeries were performed between January 2021 and December 2023.

Regarding the predominant gender, almost two-thirds of the sample were male, with 28 male patients (63.6%) and 16 female patients (36.4%). Within the diagnoses by pathology report, just over a third (38.6%) corresponded to patients with glioblastoma, and the rest had a more or less similar distribution: seven (15.9%) grade 3 astrocytomas, six (13.6%) grade 4 astrocytomas, six (13.6%) grade 3 oligodendrogliomas, six (13.6%) grade 2 astrocytomas, and two (4.5%) grade 2 oligodendrogliomas.

Regarding the IDH1 mutation status, 19 patients had the wild-type variant and 25 patients were positive for IDH1 mutation.

The surgical objective for tumor resection was the T1+C sequence in 23 patients (52.3%) and the T2-FLAIR sequence in 21 patients (47.4%).

The majority of patients had a lesion located in the frontal lobe, with a total of 30 patients (68.2%), and five patients had a lesion in the temporal lobe (11.4%), five in the insular lobe (11.4%), and four in the parietal lobe (9.1%).

The average EOR achieved using iUS as the only intraoperative aid was 81.6%. GTR was achieved in 43.2% of cases, NTR and STR were achieved in 27.3% of cases each, and a biopsy was performed in one (2.3%) case. 

Patient and tumor characteristics are summarized in Table 1.

**Table 1:** Clinical and demographic variables SD: standard deviation; GTR: gross total resection; NTR: near-total resection; STR: subtotal resection; HGG: high-grade glioma (grades 3 and 4); LGG: low-grade glioma (grades 1 and 2)

Variable	Total=44 (%)
Age in years	45.4 (19-81)
Gender	
Male	28 (63.6)
Female	16 (36.4)
Histological diagnosis	
Glioblastoma (IDH1wt)	17 (38.6)
Grade 3 astrocytoma	7 (15.9)
Grade 4 astrocytoma	6 (13.6)
Grade 3 oligodendroglioma	6 (13.6)
Grade 2 astrocytoma	6 (13.6)
Grade 2 oligodendroglioma	2 (4.5)
IDH1 mutation status	
Wild-type	17 (38.6)
Mutant	27 (61.4)
Surgical objective MRI sequence	
T2-FLAIR	21 (47.7)
T1+C	23 (52.3)
p53 mutation status	
Positive	33 (75)
Negative	11 (25)
Localization	
Frontal	30 (68.2)
Insular	5 (11.4)
Temporal	5 (11.4)
Parietal	4 (9.1)
Cortical mapping	
Yes	25 (56.8)
No	19 (43.2)
Extent of resection	
<60% biopsy	1 (2.3)
60-90% STR	12 (27.3)
90-99% NTR	12 (27.3)
100% GTR	19 (43.2)
Glioma subtype	
HGG	36 (81.8)
LGG	8 (18.2)

The statistical analysis of all the variables using the method of Spearman correlation showed that those tumors in which the surgical objective for resection was the contrast enhancement region on the T1-weighted images appeared to have greater EOR. There was no correlation between the EOR and tumor grade or molecular diagnosis. The anatomical location of the tumor and the need to use brain mapping or not did not affect the ability to achieve greater EOR either (Table 2).

**Table 2:** Correlation of clinical variables with the EOR ¥: Spearman correlation; X^2^: chi-squared; EOR: extent of resection; LGG: low-grade glioma; HGG: high-grade glioma

Variable	X^2^	p	Rho^¥^	p
LGG/HGG	11.3	0.010	0.39	0.004
IDH	14.93	0.03	-0.49	0.001
p53	0.81	0.36	-0.08	0.120
Gender	0.93	0.81	-0.111	0.236
Surgical objective	21.8	0.001	0.61	0.001
Localization	1.33	0.99	-0.001	0.993
Cortical mapping	6.14	0.01	0.37	0.012

## Discussion

Since its first use by Le Roux et al. in 1992, iUS has been used in various scenarios to aid in the resection of glial tumors [3].

It has been accepted in recent years that GTR of post-contrast T1-weighted MRI tumor in high-grade glioma and T2-FLAIR in low-grade glioma improves the OS and progression-free survival (PFS) in patients with newly diagnosed glial tumors compared to STR or biopsy in multiple large population studies [4,5]. Some retrospective studies suggest that resection of at least 70-78% of the contrast-enhancing tumor volume represents an ideal target to achieve a survival benefit [6].

Over the last couple of decades, intraoperative imaging techniques, improvements in surgical tools, and developments in monitoring techniques have improved the potential to achieve a better resection rate in glial tumors [7]. Neuronavigation systems are among the most used intraoperative tools in neurosurgery. These systems are based on preoperative imaging, allow the preoperative depiction of the lesion and surrounding anatomical structures, and guide the tumor resection [8]. The benefits of the neuronavigation system can be limited however by brain shift, in which intracerebral lesions change shape and position as a result of brain edema, gravity, lesion resection, fluid drainage, and other intraoperative factors; this shift has been estimated to be as much as 11-12 mm in some instances [9,10].

To overcome the obstacles presented by the lack of real-time navigation that the classical neuronavigation systems possess, the use of intraoperative images has emerged as a potential solution. Among these, iUS is a very attractive option, as it provides a real-time, accurate, and inexpensive imaging method for optimizing EOR in neurosurgical interventions.

iUS has been proven to be of great value in determining the volume of the lesion and its localization, especially for primary resection of gliomas and metastatic lesions [11,12]. The main issue with iUS is that it is highly user dependent, and many surgeons have encountered problems with the usage of this technology, as well as the interpretation of intraoperative US images, and this may limit its full potential. The difficulties in choosing the right probe, obtaining adequate images, and recognizing regional anatomy can be overcome by practice; training on a large number of cases is important to obtain valuable real-time information [13,14].

**Figure 2:**
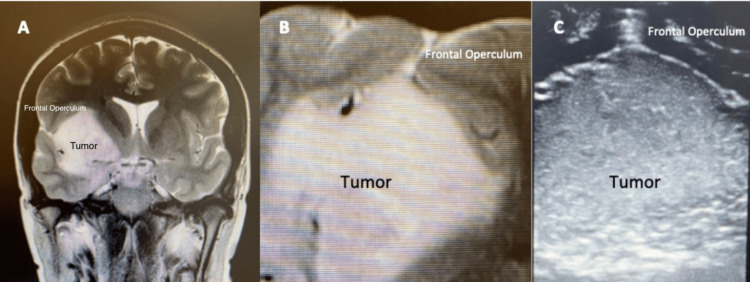
iUS images correlate with the preoperative MR images (A) Preoperative T2 coronal image depicting an insular LGG. (B) Direct correlation between the preoperative MR image and iUS image obtained during tumor localization. The hyperintense zone correlates with the hyperechoic zone in the iUS image. iUS: intraoperative ultrasound; LGG: low-grade glioma

Real-time localization of glial tumors during surgery can be achieved in up to 100% of the cases, and establishing tumor limits within the brain is the first step in achieving the greatest resection possible [15]. In our case series, we were able to identify tumor borders in all cases and found that iUS images correlate greatly with the preoperative MR images (Figure 2).

Visualization of the tumor is an important step in order to achieve a great EOR, but to be able to achieve GTR in glial tumors, several factors have to be taken into account including the surgeon's experience, the tools available in the operating room, as well as tumor characteristics like its relation to neurovascular structures, location, tumor consistency, and vascular supply.

A recent meta-analysis that included a total of 739 patients showed an average EOR of 79% in glial tumors, stating an important heterogeneity among the studies [16]. A second meta-analysis by Zhang et al. included 37 articles and established the sensibility and specificity for iUS to detect residual tumors to be about 89% and 91%, respectively [17]. In our case series, the mean EOR was 81.6%, with nearly 70% of patients having an EOR over 90%.

In our case series, tumors that had contrast enhancement in the T1 MR images had a tendency to have a greater EOR than tumors that were identified only in the T2 sequences. We believe that this may be due to the echogenic characteristics of the tumor tissue that make it more distinguishable from the healthy tissue. This may also be related to the fact the T1+C zone usually represents a lower volume for resection.

**Figure 3:**
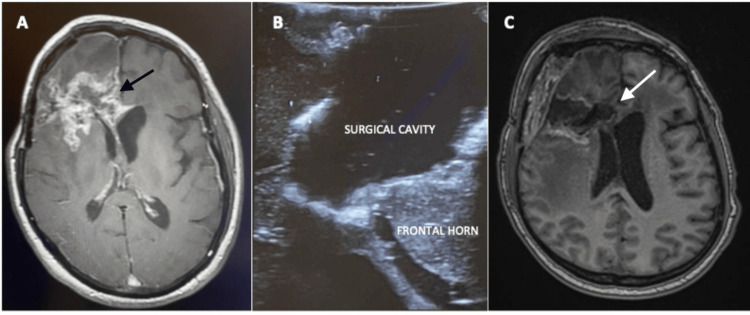
Resection of recurrent HGG (A) Preoperative T1+C image of a recurrent frontal HGG showing an irregular contrast-enhancing lesion in the right frontal lobe extending to the genu of the corpus callosum (black arrow). (B) iUS image after tumor resection. (C) Postoperative T1+C MR image showing GTR of the lesion and no visible residual contrast enhancement (white arrow). HGG: high-grade glioma; iUS: intraoperative ultrasound; GTR: gross total resection

In contrast, using iUS to estimate the EOR in glioma surgery should be interpreted with care, given that many variables could affect image quality and give false imaging as a result of acoustic enhancement artifact from hemostatic agents and clotted blood in the resection cavity, both of which can appear hyperechoic and give the impression of residual tumor [18]. In a study published by Munkvold et al., it was found that several factors could interfere with the ability of iUS to detect residual tumors during glioma surgery. They determined that tumor volume and tumor depth were the main factors that influenced the sensitivity of iUS to detect residual tumors, with small superficial tumors being more likely to be completely resected [19]. Figure 3 shows the resection of recurrent high-grade glioma.

The advances in technology have improved the capacity of iUS to better detect and differentiate tumor tissue from healthy tissue. The developments of new US transducers, contrast agents, and processing systems have helped to overcome the previous limitations of iUS. Contrast-enhanced US involves the injections of microbubbles that comprise an inert gas, such as perfluorocarbon or nitrogen, encapsulated in a layer of protein or polymers. These microbubbles are able to cross into the arterial circulation, acting as a contrast agent depending on the tumor vascularity and perfusion [20]. Several studies have demonstrated improved visualization and delineation of tumor boundaries versus standard B-mode, and even more importantly, it has been proven helpful in detecting residual tumors following resection in high-grade gliomas, with greater sensitivity compared to conventional B-mode iUS [21]. Contrast-enhanced US has its limitations mainly because of the learning curve that is needed at the beginning. Also, its sensitivity can be altered in tumors with low vascularity and previously treated tumors [22].

Strain elastography is another feature of US technology that evaluates tissue macrostructure, as it compares characteristics of the ultrasound beam through tissue before and after compression, and so it is able to map tissue stiffness. Using this technology in combination with B-mode US, a better differentiation of tumor and normal brain tissue can be achieved [23,24]. Unfortunately at the present time, elastography remains in the research realm, with its role in neuro-oncology remaining uncertain.

The retrospective nature of the study and the size of the sample are limitations that this study possesses and should be taken into account when interpreting the results. 

## Conclusions

It is possible to achieve an adequate EOR in glioma surgery utilizing iUS as the only intraoperative imaging aid, and the data shows that neuronavigation systems are not essential to achieve these results.

iUS by itself has been proven to be a valuable tool to improve the localization and resection of supratentorial gliomas. Given its versatility and cost-effectiveness, iUS should be a part of the intraoperative neurosurgical tools in the field of neuro-oncology.

The widespread adoption of iUS has been limited by a perceived steep learning curve; however, this can be overcome by proper training in order to understand the basics of US and the proper technique to obtain the benefits of this technology.
